# Dental Education and Oral Health Service in Iraq

**Published:** 2017-05

**Authors:** Ammar NH ALBUJEER, Abbas TAHER

**Affiliations:** 1. Dept. of Oral and Maxillofacial Surgery, School of Medicine and Dentistry, Santiago de Compostela University, Santiago de Compostela, Spain; 2. Nab’a Al-Hayat Foundation for Medical Sciences and Health Care, Najaf, Iraq; 3. Dept. of Oral and Maxillofacial Surgery, Faculty of Dentistry, University of Kufa, Kufa, Iraq

## Dear Editor-in-Chief

As we know all evidence-based information have linked between Oral Health and Systemic disease (1, 2). All the oral health problems have a great impact on many sectors, personal, social, and government from economic & health wise view in developing and non-developing countries (3). One of these countries is Iraq. The oral health services in Iraq are provided by the Dental Schools, National Health Service (NHS), and private sectors. Most of the Iraqi dental professions were graduated from Iraqi dental faculties. The dental services in Iraq are under control of the government according to the Iraqi constitution (4).

In 2010, there was more than 5900000 dental visits in the primary dental care of the NHS. The treatment was for securing the oral health including, prevention, restoration, prosthodontics, orthodontic & surgical treatments. The cost of the treatment provided by the MOH is very cheap and about half USD per course of treatment (50 cent) (5).

The oral health service was developed significantly in Iraq after 2003. Before 2003, the number of registered dentists was less than 3000 according to Iraqi Dental Association (IDA) records. In 2010, there were 4863 dentists with population ratio 1.7/10000 (5). In 2015, the number of IDA registered members has risen to 7277 dentists with population ratio 2.3/10000 (6).

Currently there are more than 8500 registered dentists, with the dentist population ratio of 2.6 dentist for every 10000 citizens. This number will be gradually increased because of the new establishment of the dental schools (Government & Private). This will lead to increase the number of dental school graduates, and may be causing economic problem to the MOH & MOHE, because all the medical science graduates are by law should be employed by the government according to article 6 for 2000 of the Ministry of Health. The estimated number to be achieved in year 2020 is 6.4 Dentist toward 10000. Most of those dentists who are working in MOH & MOHE are working at the private sectors in the afternoon. About 10% of them are working at the universities government and private sectors.

One of the major problems in Iraq is the shortage in the Middle Dental Staffs (Dental Nurses, Hygienist, Dental Oral Hygiene educationist, Dental Technicians). We do hope the Government will take into consideration this shortage seriously and open some new educational centers (Government or private for this purposes).
